# Upregulation of the Glutaminase II Pathway Contributes to Glutamate Production upon Glutaminase 1 Inhibition in Pancreatic Cancer

**DOI:** 10.1002/pmic.201800451

**Published:** 2019-08-01

**Authors:** Sunag Udupa, Stephanie Nguyen, Giang Hoang, Tu Nguyen, Addison Quinones, Khoa Pham, Ryoichi Asaka, Kiet Nguyen, Cissy Zhang, Amira Elgogary, Jin G. Jung, Qingguo Xu, Jie Fu, Ajit G. Thomas, Takashi Tsukamoto, Justin Hanes, Barbara S. Slusher, Arthur J. L. Cooper, Anne Le

**Affiliations:** ^1^ Department of Pathology Johns Hopkins University School of Medicine Baltimore MD 21205 USA; ^2^ Department of Ophthalmology and Wilmer Eye Institute Center for Nanomedicine Johns Hopkins University School of Medicine Baltimore MD 21205 USA; ^3^ Johns Hopkins Drug Discovery Johns Hopkins University School of Medicine Baltimore MD 21205 USA; ^4^ Department of Neurology Johns Hopkins University School of Medicine Baltimore MD 21205 USA; ^5^ Department of Oncology Johns Hopkins University School of Medicine Baltimore MD 21205 USA; ^6^ Department of Biomedical Engineering Johns Hopkins University School of Medicine Baltimore MD 21205 USA; ^7^ Department of Pharmacology and Molecular Sciences Johns Hopkins University School of Medicine Baltimore MD 21205 USA; ^8^ Department of Medicine Johns Hopkins University School of Medicine Baltimore MD 21205 USA; ^9^ Department of Psychiatry Johns Hopkins University School of Medicine Baltimore MD 21205 USA; ^10^ Department of Chemical and Biomolecular Engineering Johns Hopkins University Whiting School of Engineering Baltimore MD 21218 USA; ^11^ Department of Biochemistry and Molecular Biology New York Medical College Valhalla NY 10595 USA; ^12^ Department of Pharmaceutics Virginia Commonwealth University Richmond VA 23298 USA

**Keywords:** glutaminase 1 inhibition, glutaminase II pathway, glutamine transaminase K

## Abstract

The targeting of glutamine metabolism specifically via pharmacological inhibition of glutaminase 1 (GLS1) has been translated into clinical trials as a novel therapy for several cancers. The results, though encouraging, show room for improvement in terms of tumor reduction. In this study, the glutaminase II pathway is found to be upregulated for glutamate production upon GLS1 inhibition in pancreatic tumors. Moreover, genetic suppression of glutamine transaminase K (GTK), a key enzyme of the glutaminase II pathway, leads to the complete inhibition of pancreatic tumorigenesis in vivo unveiling GTK as a new metabolic target for cancer therapy. These results suggest that current trials using GLS1 inhibition as a therapeutic approach targeting glutamine metabolism in cancer should take into account the upregulation of other metabolic pathways that can lead to glutamate production; one such pathway is the glutaminase II pathway via GTK.

The reprogramming of metabolism is one of the key hallmarks of cancer. Alterations in metabolism provide cancers with the ability to proliferate and resist metabolic deprivation.^1^, ^2^ We, and others, have established that glutamine metabolism plays a critical role in cancer cell proliferation, providing a number of clinically relevant metabolic targets for the treatment of cancer.^3^, ^4^ CB‐839, a small molecule inhibitor of glutaminase 1 (GLS1), an enzyme that converts glutamine to glutamate, has recently reached clinical trials as an avenue for cancer therapy.^5^ However, GLS1 inhibition monotherapy by CB‐839 has resulted in limited clinical efficacy and only partial tumor reduction in mice bearing orthotopic pancreatic tumors in vivo.^1^ Thus, we sought to improve the therapeutic index of GLS1 inhibition by understanding how tumors compensate for the loss of GLS1 activity. Global metabolic profiling of pancreatic cells in vitro upon GLS1 inhibition did not identify specific pathways involved in glutamate production upon GLS1 inhibition.^6^ Moreover, a recent study showed cancer cells to have different metabolic behaviors in culture as compared to in vivo tumors, especially in regards to glutamine metabolism, thereby highlighting the importance of studying cancer metabolism in the tumor microenvironment.^7^

In the present study, we employed mass‐spectrometry‐based stable isotope‐resolved metabolomics (SIRM) with ^13^C_5_^15^N_2_‐labeled‐glutamine to identify which specific metabolic pathways pancreatic tumors utilize upon GLS1 inhibition in vivo. Following pharmacological inhibition of GLS1 by bis‐2‐(5‐phenylacetamido‐1,3,4‐thiadiazol‐2‐yl)ethyl sulfide (BPTES) encapsulated in nanoparticles (BPTES‐NP) to treat patient‐derived pancreatic ductal adenocarcinoma (PDAC) orthotopic tumors,^1^ we injected mice with ^13^C_5_^15^N_2_‐glutamine (labeled glutamine with an increase in molecular mass of 7 mass units relative to unlabeled ^12^C_5_^14^N_2_‐glutamine) in order to trace glutamine metabolism. BPTES‐NP treatment was previously shown to decrease PDAC orthotopic tumor weights in mice.^1^ Surprisingly, we noticed an increase in (m+5) glutamate in BPTES‐NP‐treated tumors as compared to the blank nanoparticle vehicle control (Blank‐NP) (**Figure** 1A,B and Figure S1A, Supporting Information). GLS1 facilitates the direct conversion of ^13^C_5_^15^N_2_‐glutamine (m+7) into ^13^C_5_^15^N_1_‐glutamate (m+6) and ammonia. However, the increased production of (m+5) glutamate isotopologue upon GLS1 inhibition suggested an alternative pathway for glutamate production beyond glutaminolysis. Due to the ^13^C_5_^15^N_2_‐glutamine label, the observed (m+5) glutamate must be either ^13^C_4_^15^N_1_‐glutamate or ^13^C_5_‐glutamate. The formation of ^13^C_4_^15^N_1_‐glutamate requires the loss of one ^13^C_1_ and one ^15^N_1_ label from ^13^C_5_^15^N_2_‐glutamine which is very unlikely. In regards to the formation of ^13^C_5_‐glutamate, this (m+5) glutamate could be made via transamination of ^13^C_5_‐α‐ketoglutarate (m+5) derived from ^13^C_5_^15^N_1_‐glutamate (m+6). In order for this pathway to occur, this ^13^C_5_^15^N_1_‐glutamate (m+6) would be made from ^13^C_5_^15^N_2_‐glutamine (m+7) via GLS1 (Figure S1B, Supporting Information). In the present study, mice were treated with the GLS1 inhibitor BPTES‐NP. Therefore, while ^13^C_5_‐glutamate (m+5) could be made through the transamination of ^13^C_5_‐α‐ketoglutarate (m+5) originating from ^13^C_5_^15^N_1_‐glutamate (m+6), this pathway would result in a decrease of (m+5) glutamate in tumors treated with the BPTES‐NP as compared to tumors receiving the vehicle control. However, we observed an increase in (m+5) glutamate upon GLS1 inhibition in vivo as compared to vehicle control‐treated tumors. Therefore, this (m+5) glutamate must be from a source other than from transamination of ^13^C_5_‐α‐ketoglutarate (m+5) derived from ^13^C_5_^15^N_1_‐glutamate (m+6).

**Significance Statement**

Here, we report an increase in glutamate production via the glutaminase II pathway upon GLS1 inhibition treatment in patient‐derived orthotopic pancreatic cancer in vivo. This finding highlights the adaptive ability of cancers to readjust their metabolic network in order to survive metabolic deprivation. We also identified glutamine transaminase K (GTK), a key enzyme of the glutaminase II pathway, as a metabolic target for cancer therapy given that genetic suppression of this enzyme leads to complete inhibition of pancreatic tumorigenesis in vivo. A pharmacotherapeutic approach for pancreatic cancer treatment via the glutamine antagonist prodrug, JHU083 (ethyl 2‐(2‐amino‐4‐methylpentanamido)‐DON), was also investigated as a proof‐of‐concept for improving therapeutic efficacy by blocking global metabolic pathways from glutamine utilization. The results suggest that a more targeted approach, specifically by combination of GLS1 and GTK inhibition, is required for cancer therapy.

**Figure 1:**
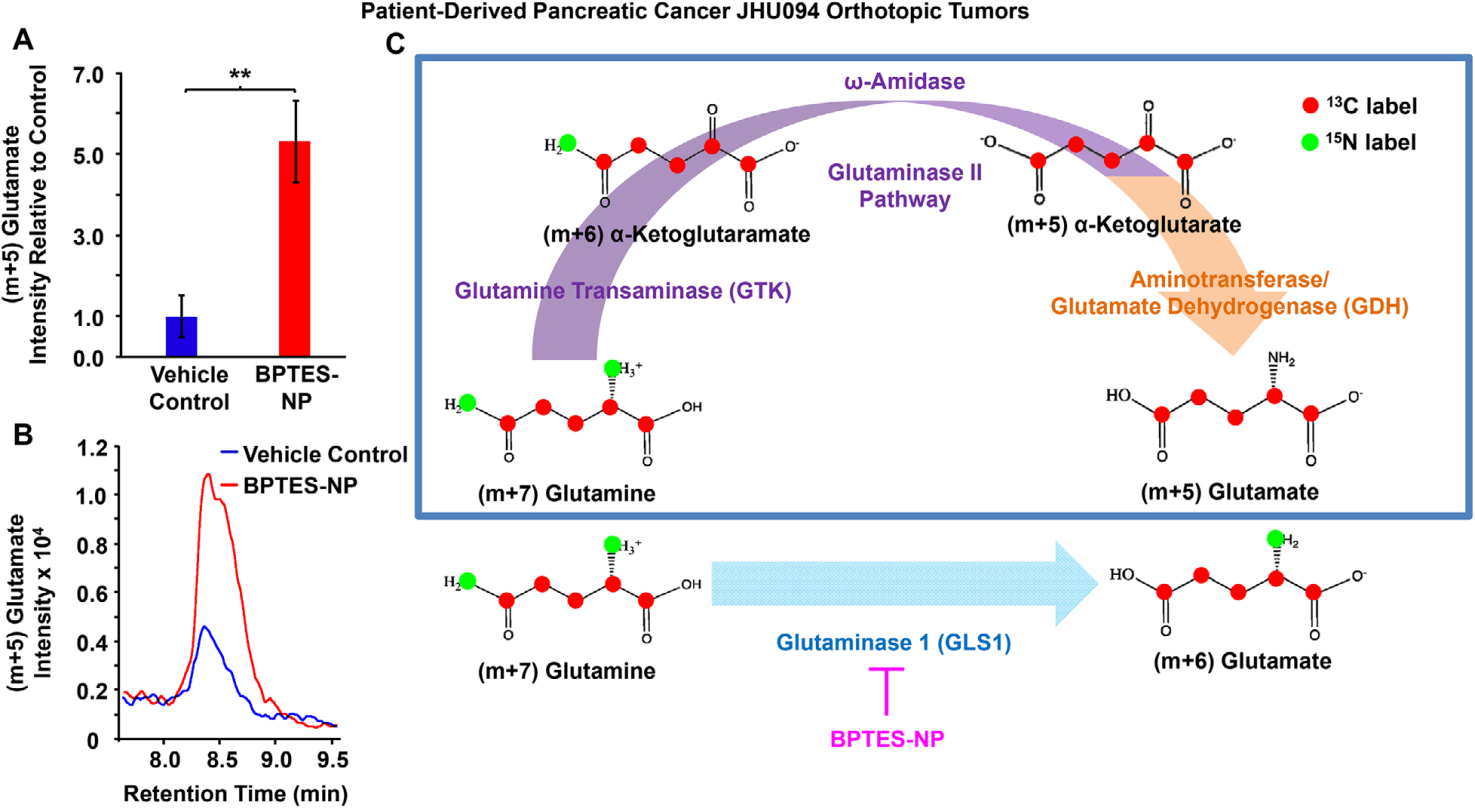
Increased (m+5) glutamate in patient‐derived pancreatic cancer (JHU094) orthotopic tumors after treatment with GLS1 inhibitor, BPTES‐NP. Patient‐derived pancreatic tumors were surgically implanted into the pancreas of mice to generate orthotopic tumors. Four weeks after orthotopic tumor implantation, mice received either GLS1 inhibitor, 54 mg kg^−1^ BPTES‐NP, or vehicle control, Blank‐NP, via intravenous injection every 3 days for 16 days. Tumors were then excised and subjected to metabolomics analysis. A,B) Comparison of (m+5) glutamate intensities between vehicle control and BPTES‐NP‐treated tumors. (A) Intensity relative to vehicle control and (B) extracted ion chromatograms of (m+5) glutamate isotopologue in patient‐derived pancreatic orthotopic tumors treated with vehicle control, shown in blue, or BPTES‐NP, shown in red. Ion chromatograms are shown as the peak area denoting the total signal intensity of (m+5) glutamate in the respective tumor groups. All data is shown as mean ± SEM (*n* = 6 per group). This experiment was replicated twice with similar results. **p < 0.01 (Student's *t*‐test) where indicated. C) Illustration of the formation of (m+5) glutamate via the glutaminase II pathway coupled to an α‐ketoglutarate‐linked aminotransferase or the glutamate dehydrogenase (GDH) reaction. The glutaminase II pathway (purple arrow) consists of the GTK catalyzed conversion of glutamine to α‐ketoglutaramate (KGM) followed by hydrolysis of KGM to α‐ketoglutarate by ω‐amidase. This pathway then acts as a source of glutamate via transamination of α‐ketoglutarate by an α‐ketoglutarate‐linked aminotransferase or by reductive amination catalyzed by glutamate dehydrogenase (GDH) (orange arrow). For comparison, the glutaminase 1 (GLS1) reaction for the formation of (m+6) glutamate from (m+7) glutamine (blue arrow) and GLS1 inhibition by BPTES‐NP (pink arrow) are shown. Enzymes are shown in purple (glutaminase II pathway), orange (α‐ketoglutarate‐linked aminotransferase or GDH), or blue (GLS1). Metabolites are shown in black. Red circles indicate ^13^C labeling, and green circles indicate ^15^N labeling.

The formation of ^13^C_5_‐glutamate is explained through the transamination of α‐ketoglutarate derived from the glutaminase II pathway (Figure 1C, purple arrow; Equations (1) and (2)), into ^13^C_5_‐glutamate. The glutaminase II pathway consists of the glutamine transaminase K (GTK) (or kynurenine aminotransferase I^8^, ^9^) catalyzed conversion of glutamine to α‐ketoglutaramate (KGM) using a suitable α‐keto acid acceptor^10^, ^11^, ^12^ (Equation (1)) followed by hydrolysis of KGM to α‐ketoglutarate catalyzed by ω‐amidodicarboxylate amidohydrolase (ω‐amidase)^13^ (Equation (2)). This pathway can then act as a source of glutamate via transamination of α‐ketoglutarate by an α‐ketoglutarate‐linked aminotransferase (Equation (3)) or by reductive amination catalyzed by glutamate dehydrogenase (GDH)^14^ (Figure 1C, orange arrow).

Glutaminase II Pathway

(1) $$\begin{array}{c} (m\;+\;7)\;L-\mathrm{glutamine}\;+\;\alpha \text{-keto}\;\text{acid}⇆\mathrm{KGM}\;+\;\mathrm{L-amino}\;\text{acid} \end{array}$$

(2) $$\mathrm{KGM}+H_{2}O\to \alpha -\mathrm{ketoglutarate}+N{H_{4}}^{+}$$

Transamination Reaction

(3) $$\begin{array}{ccc} & & \alpha -\mathrm{ketoglutarate}+\mathrm{L-amino}\;\text{acid}⇆\left(m+5\right)\text{L-glutamate}\\ & & \;\;\;\;+\;\alpha -\;\text{keto}\;\text{acid} \end{array}$$

Glutamate generated from glutamine through the glutaminase II pathway (Equations (1) and (2)) coupled to an α‐ketoglutarate‐linked aminotransferase (Equation (3)) or to GDH explains the loss of both ^15^N‐labels from glutamine (Figure 1C, blue box). Of note, l‐amino acids, such as l‐aspartate in the transamination reaction only provide the amine group of glutamate.^15^ The carbon backbone of glutamate is derived from α‐ketoglutarate (Figure S1B, Supporting Information).^15^, ^16^ Therefore, the glutaminase II pathway is the most probable source of the observed (m+5) glutamate found to be present in the surviving tumors.

We then sought to identify increases in the glutaminase II pathway intermediates, specifically KGM, in order to confirm that this pathway is a source of the observed increase in (m+5) glutamate production. It is known that KGM reversibly cyclizes to 2‐hydroxy‐5‐oxoproline and, at equilibrium, mainly exists in this cyclic form at physiological pH.^17^ In addition, only the open form of KGM is a substrate of ω‐amidase,^17^ the enzyme responsible for the hydrolysis of KGM into α‐ketoglutarate. Thus, we used both mass spectrometry and ^1^H NMR to assess intratumoral KGM levels, but we were only able to accurately identify KGM structure using ^1^H NMR. The data did indeed confirm an increase in KGM in BPTES‐NP‐treated tumors as compared to Blank‐NP‐treated tumors (**Figure** 2A–C). KGM being the product of glutamine transamination by GTK,^13^, ^18^ we next investigated the role of GTK, a major enzyme in the glutaminase II pathway, in catalyzing glutamine conversion to KGM, the carbon skeleton of which is eventually incorporated into glutamate to promote cancer growth. The previous findings that KGM is present in body fluids and organ tissues, indicate that the glutamine transaminases are active in vivo.^19^ Moreover, the results of glutamine tracer studies suggest that humans have a large capacity to transaminate glutamine.^20^ In order to assess the contribution of GTK in promoting cancer growth, we first assessed the expression of GTK in eight patient‐derived pancreatic cancer cell lines and identified P198 cells to have the strongest expression of GTK among these cell lines (**Figure** 3A). We then knocked‐down (KD) the expression of GTK in P198 cells using short‐hairpin RNA (shRNA) lentivirus carrying the shGTK vector (Figure 3B) and found that P198 shGTK‐KD cells exhibited lower cell numbers than P198 shControl cells (Figure 3C). This data suggests that GTK plays an important role in cancer cell proliferation. Importantly, P198 shGTK‐KD cells treated with a GLS1 inhibitor, BPTES, were observed to have the lowest cell numbers among all the groups (Figure 3C), demonstrating that genetic targeting of GTK in combination with pharmacological inhibition of GLS1 accentuates cancer cell growth inhibition in vitro. However, as mentioned above, glutamine metabolism of cancer cells in vitro has been demonstrated to be different than that in in vivo tumors.^7^ Therefore, in order to further investigate the role of GTK in cancer, we translated our study into an in vivo model. We used P198 shGTK‐KD and P198 shControl cells to generate xenograft tumors and observed that shGTK‐KD completely prevented tumor formation while shControl tumors grew exponentially (Figure 3D**)**. These findings suggest that the genetic suppression of GTK has a profound effect on the abolishment of tumorigenesis of pancreatic cancer and reveal GTK as a metabolic target for cancer therapy. Of note, due to the absence of P198 shGTK‐KD tumors in vivo, there was no tumor tissue available for metabolomics analysis.

**Figure 2:**
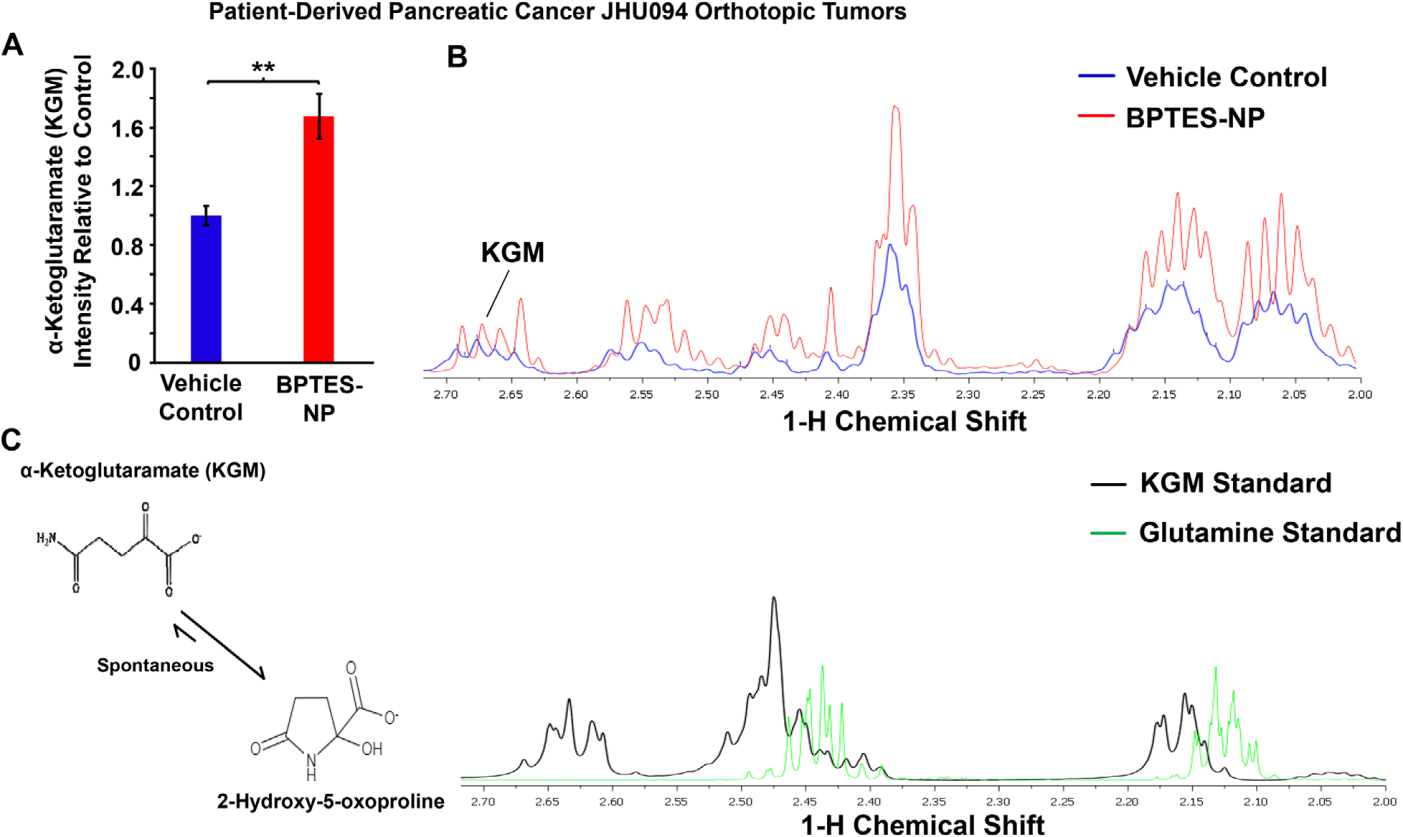
Metabolomics analysis of α‐ketoglutaramate (KGM) in patient‐derived pancreatic cancer (JHU094) orthotopic tumors treated with BPTES‐NP. A–C) Comparison of KGM intensities between vehicle control and BPTES‐NP‐treated tumors. (A) Intensity relative to vehicle control and (B) ^1^H NMR spectra of KGM in patient‐derived pancreatic orthotopic tumor homogenate treated with vehicle control, shown in blue, or BPTES‐NP, shown in red. (C) Metabolite standard spectra are shown with KGM shown in black, and glutamine shown in green. Due to the equilibrium of KGM with the cyclic lactam form (2‐hydroxy‐5‐oxoproline (structures shown in panel (C)), the hydrogens attached to the carbons 3 and 4 are in different chemical environments. All data is shown as mean ± SEM (*n* = 8 per group). **p < 0.01 (Student's *t*‐test) where indicated.

**Figure 3:**
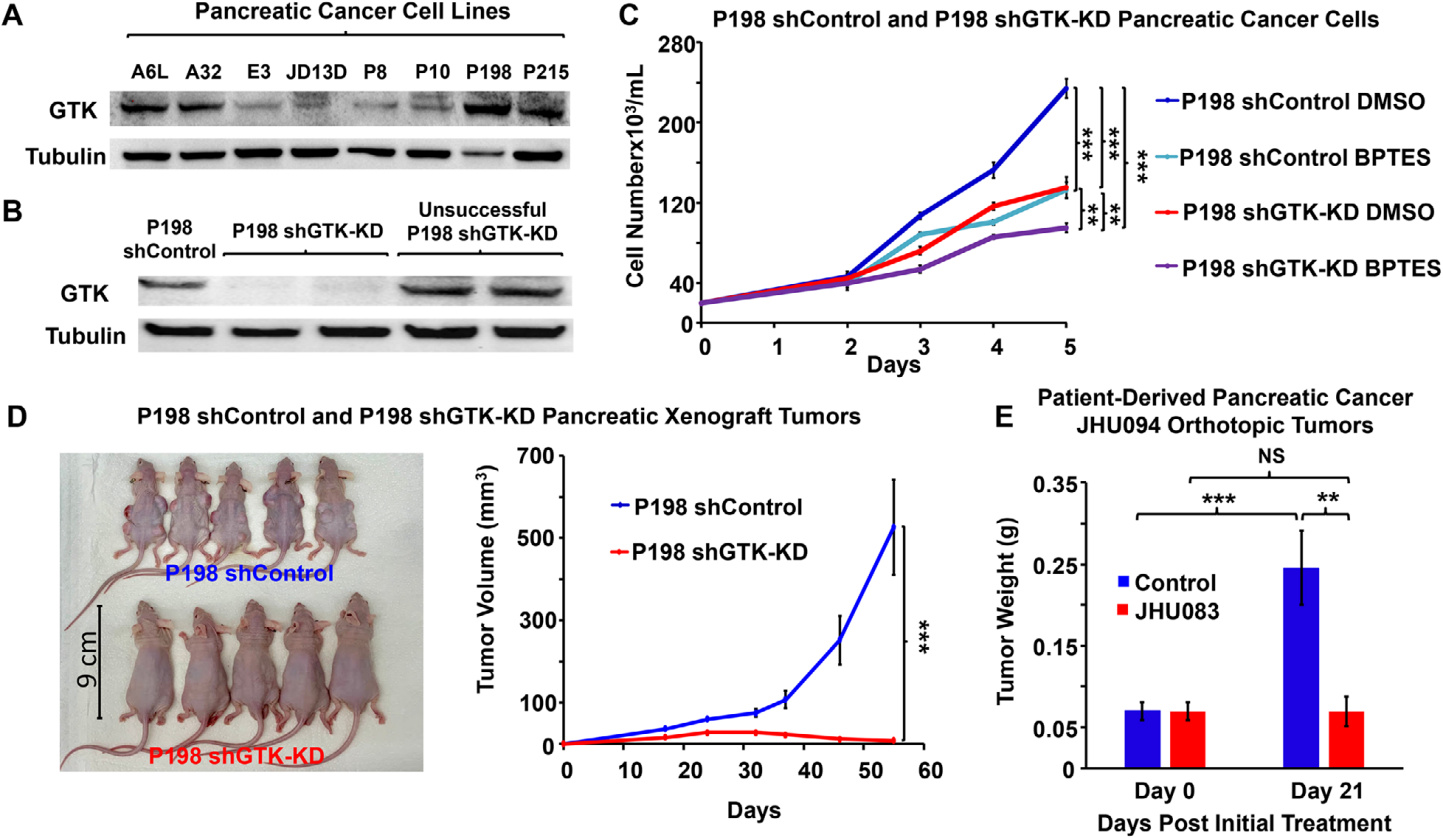
Effect of targeting the glutaminase II pathway in pancreatic cancer. A,B) Assessment of GTK expression in pancreatic cancer cells. GTK expression was assessed by western blotting in (A) A6L, A32, E3, JD13D, P8, P10, P198, and P215 pancreatic cancer cell lines, (B) P198 shGTK‐KD, and P198 shControl. Tubulin served as a loading control. C) Effect of glutaminase 1 inhibitor, BPTES, on cell numbers of P198 shGTK‐KD and P198 shControl in vitro. Pancreatic cancer cells P198 shControl and P198 shGTK‐KD were grown in an incubator at 37 °C in 5% CO_2_ and 95% air v/v in DMEM containing 10% FBS, 1% penicillin‐streptomycin, and 1 µg mL^−1^ puromycin. Cells were treated with DMSO vehicle control or 10 µm BPTES. Cell numbers were assessed at 48, 72, 96, and 120 h after treatment (*n* = 3 samples per group and per time point). This experiment was replicated three times with similar results. D) P198 shGTK‐KD and P198 shControl Xenograft Tumors. 5 × 10^6^ P198 shGTK‐KD and P198 shControl cells were subcutaneously injected into the backs of mice (*n* = 20 tumors per group and per time point). Tumor formation and size progression were measured over 52 days and tumor sizes in the mice at day 52 are pictured. E) Effect of glutamine antagonist, JHU083 (1 mg kg^−1^ 5 days a week for 3 weeks) on patient‐derived pancreatic cancer (JHU094) orthotopic tumors. Patient‐derived pancreatic cancer (JHU094) tumors were implanted into the pancreas of mice. Tumor weights of Day 0 (the starting treatment day) were assessed from 12 mice that were euthanized at Day 0 and their tumors were extracted from the pancreas and weighed. Mice received 1 mg kg^−1^ (0.022 mg of JHU083 in 100 µL of vehicle control per mouse) of JHU083, by intraperitoneal injection (IP) or 100 µL of vehicle control 5 days per week for 3 weeks. The vehicle control consisted of 95% v/v HEPES buffered saline in ethanol. Tumors were then excised and weighed. All values are shown as mean ± SEM (*n* = 20 tumors per group for every time point for xenograft tumors, 10 per group for JHU083‐treated tumors). NS, not significant, *p < 0.05, **p < 0.01, ***p < 0.001 (Student's *t*‐test) where indicated.

These results suggest that current trials using GLS1 inhibition as a therapeutic approach targeting glutamine metabolism in cancer should take into account other metabolic pathways that can upregulate glutamate production such as the glutaminase II pathway via GTK. Thus, we next sought to investigate the pharmacotherapeutic potential of GTK inhibition for cancer therapy. However, given that inhibitors of GTK are not commercially available to combine with pharmacological GLS1 inhibition, an attempt to use glutamine antagonism was explored as proof‐of‐concept for improving therapeutic efficacy by blocking global metabolic pathways from glutamine utilization. Thus, we employed our previously reported global glutamine antagonist, termed JHU083 (ethyl 2‐(2‐amino‐4‐methylpentanamido)‐DON), a prodrug of 6‐diazo‐5‐oxo‐l‐norleucine (DON).^21^, ^22^ We generated patient‐derived pancreatic orthotopic tumors (JHU094), the same tumors used in our initial in vivo metabolomics analysis above, which were found to closely mimic the molecular, pathobiological, and clinical characteristics of PDAC.^23^ The mice were then treated with 1 mg kg^−1^ JHU083 or vehicle control five times per week for 3 weeks via intraperitoneal (IP) injection. After 3 weeks of treatment, while tumor weights of vehicle control mice increased significantly, JHU083 treatment completely inhibited tumor growth (Figure 3E). Although the assessment of hematology, liver, and kidney function showed acceptable ranges in mice after JHU083 treatment (Figure S2, Supporting Information), two of the JHU083‐treated mice did not survive the first week of dosing for unknown causes. Taken together, the newly discovered upregulation of glutamate production via the glutaminase II pathway in cancer upon GLS1 inhibition and the off‐target effects of global glutamine antagonism strongly suggest a need for the development of a specific GTK inhibitor to combine with GLS1 inhibition for cancer therapy. Of note, due to the location of pancreatic orthotopic tumors, we were unable to measure tumor volume over time. Also, patient‐derived pancreatic orthotopic tumors’ sizes often exceed the allowable limit of Johns Hopkins University Animal Care and Use Committee before causing death. Therefore, data on the life span, although it could be evidence of tumor regression, was not achievable. Moreover, the inhibitory effect of pharmacological inhibition of glutamine metabolism by JHU083 on tumor growth was not as pronounced as that observed with genetic suppression of GTK as the tumors had already formed prior to JHU083 treatment. We acknowledge that there are other pathways that can produce glutamate. Glutamate can be produced, for example, through Δ^1^‐pyrroline‐5‐carboxylate (P5C) derived from the amino acids proline and ornithine.^24^ However, a recent study of melanoma has reported that intracellular P5C is rapidly converted to proline and thus glutamate production from this source is limited.^25^, ^26^ Alternatively, the metabolite *N*‐acetylaspartylglutamate has been shown to produce glutamate in pancreatic cancer both in vitro and in vivo via glutamate carboxypeptidase II (GCPII)‐catalyzed hydrolysis.^27^

Therapeutic approaches targeting glutamine metabolism have been predominantly focused on inhibiting the glutaminase‐catalyzed conversion of glutamine into glutamate and ammonia. In particular, with regard to the overexpression of the kidney isozyme of glutaminase, GLS1, in a large number of cancers^28^, ^29^ including PDAC, a number of GLS1 inhibitors, including CB‐839 and BPTES‐NP, have been developed and CB‐839 has even reached clinical trials.^5^ The clinical efficacy, however, shows opportunities for improvement in regards to tumor reduction. In the current study, with the use of SIRM and ^1^H NMR, we identified the glutaminase II pathway coupled to an α‐ketoglutarate‐linked aminotransferase or GDH to be a key metabolic route utilized by tumors to produce glutamate with increased levels of (m+5) glutamate and KGM upon GLS1 inhibition.

The glutaminase II pathway has been studied in many hyperammonemic diseases of the liver and urea cycle.^18^, ^30^, ^31^, ^32^, ^33^ However, the role of the glutaminase II pathway in cancer has been largely overlooked thus far.^13^, ^34^ In the present study, we found GTK, an intermediary enzyme of the glutaminase II pathway, to play a vital role in pancreatic tumorigenesis. Specifically, genetic suppression of GTK inhibited tumor formation in vivo and thus this enzyme provides a promising target for the development of selective GTK inhibitors to combine with existing GLS1 inhibitors for the treatment of pancreatic cancer.

In summary, the uncovering of the role of the glutaminase II pathway as a source of the carbon backbone of glutamate upon single‐therapy GLS1 inhibition unveils the therapeutic potential of the glutaminase II pathway for the treatment of pancreatic cancer. This metabolic adaptability of tumors explains the current outcomes of single‐targeted GLS1 inhibition in pancreatic cancer and justifies the need for a more strategic approach, specifically by the combination of GLS1 and GTK inhibition, aiming at both main and adaptive metabolic networks.

## Experimental Section

Detailed materials and methods are available online in the Supporting Information.

##### Animal Studies and Drug Treatment In Vivo

For targeting the glutaminase 1 pathway, mice bearing patient‐derived pancreatic orthotopic tumors received 54 mg kg^−1^ of BPTES‐NP, 1.2 mg BPTES in 100 µL of nanoparticles per mouse, or 100 µL of blank‐nanoparticles (Blank‐NP) as vehicle control.

For targeting global glutamine metabolism, mice bearing patient‐derived pancreatic orthotopic tumors received 1 mg kg^−1^ of JHU083, 0.022 mg of JHU083 in 100 µL of vehicle control per mouse, or 100 µL of vehicle control. The vehicle control consisted of 95% v/v HEPES buffered saline in ethanol.

For investigation of the role of GTK in vivo, 5 × 10^6^ P198 shGTK‐KD or shControl cells were suspended in a 50% v/v matrigel and DMEM mixture and were injected subcutaneously into male Foxn1^nu^ athymic nude mice per tumor per site. Tumor formation and size progression were monitored over 52 days. Our animal study protocol to study patient‐derived orthotopic pancreatic cancer was approved by Johns Hopkins University Animal Care and Use Committee. Thereby, the protocol is compliant with the Association for Assessment and Accreditation of Laboratory Animal Care guideline.

##### Metabolomics Analysis of Patient‐Derived Orthotopic Tumors In Vivo

At the end of the BPTES‐NP efficacy experiment, mice were injected via IP administration with 100 µL of 100 mm sterile‐filtered ^13^C_5_^15^N_2_‐glutamine (m+7) in order to trace glutamine metabolism. Metabolomics analysis was then conducted using SIRM and ^1^H NMR. The α‐ketoglutaramate (KGM) used for the ^1^H NMR study was synthesized by the method of Krasnikov et al.^35^

##### Lentiviral Transduction of Pancreatic Cancer P198 Cells

Pancreatic cancer P198 cells were plated at a density of 20 000 cells mL^−1^ in DMEM containing 10% v/v FBS and 1% v/v penicillin–streptomycin at 37 °C in 5% CO_2_ and 95% air v/v. The cells were then transfected with lentivirus carrying the shGTK vector or shControl vector at a concentration of 400 000 transducing units per milliliter in the presence of 2 µg mL^−1^ polybrene.

##### Pancreatic Cancer P198 Drug Treatment In Vitro

Pancreatic cancer P198 shGTK‐KD and P198 shControl cells were plated in multiple 24 well plates at a density of 20 000 cells mL^−1^ in DMEM containing 10% v/v FBS, 1% v/v Pen–Strep, and 1 µg mL^−1^ puromycin and left to adhere overnight in an incubator at 37 °C in 5% CO_2_ and 95% air v/v. The following day, cell groups were treated with 10 µm BPTES in dimethyl sulfoxide (DMSO) or DMSO vehicle control. Cell number and viability were assessed using a Vi‐Cell XR Cell Viability Analyzer.

##### Western Blot Analysis

Pancreatic cancer P198 shGTK‐KD and shControl cells were lysed to extract proteins for protein concentration. These proteins were used for western blotting as described in the Supporting Information. The protein expression image was subsequently developed using a ChemiDoc XRS+ Imaging System.

##### Statistical Analysis

Values are reported as mean ± SEM. Statistical significance was determined as NS, not significant, *p < 0.05, **p < 0.01, ***p < 0.001 using the Student's *t‐*test. Experiment‐specific statistical details, including the number of samples per experiment, can be found in the accompanying figure legends.

## Conflict of Interest

JHU083 and other DON prodrugs have been licensed to Dracen Pharmaceuticals. Under the license agreement between Dracen Pharmaceuticals, Inc. and the Johns Hopkins University, author B.S.S. is entitled to royalty distributions. B.S.S. is also a founder of and holds equity in Dracen Pharmaceuticals. These arrangements have been reviewed and approved by the Johns Hopkins University and the IOCB in accordance with institutional conflict of interest policies. All other authors declare no conflict of interest.

## Supporting information

Supporting Information

Supporting Information

Supporting Information
