# Supplementary material for: Upregulation of the Glutaminase II Pathway Contributes to Glutamate Production upon Glutaminase 1 Inhibition in Pancreatic Cancer
Source: Proteomics. 2019 Aug 1;19(21-22):1800451. doi: 10.1002/pmic.201800451 (PMC6851409; doi:10.1002/pmic.201800451)
Supplement: Supplementary file 1 — Supporting Information [file PMIC-19-na-s003.pptx]

## Slide 1
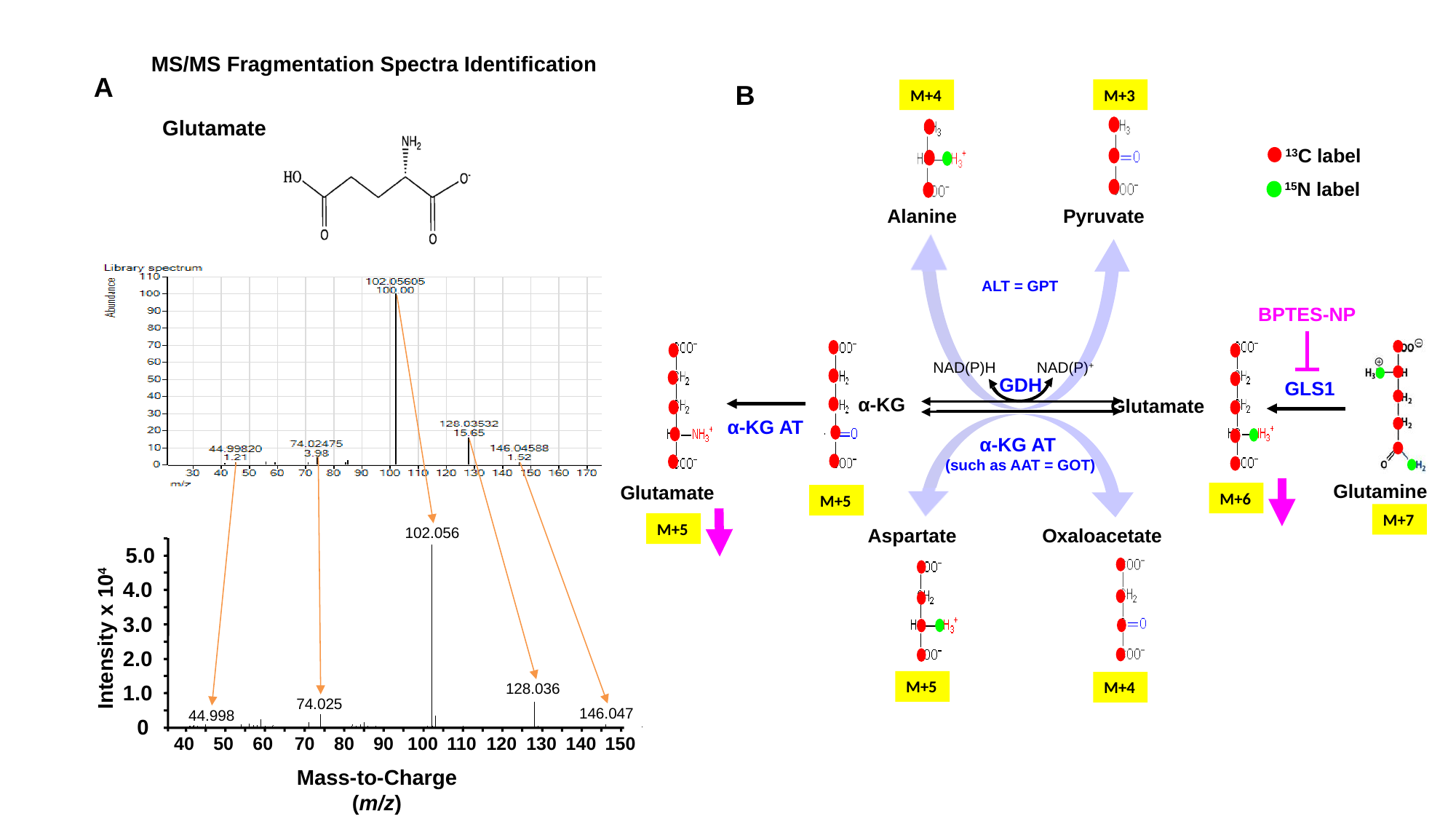

MS/MS Fragmentation Spectra Identification
102.056
5.0
4.0
3.0
2.0
1.0
0
128.036
74.025
146.047
44.998
40
50
60
70
80
90
100
110
120
130
140
150
Intensity x 104
Mass-to-Charge (m/z)
Glutamate
A
B
M+3
M+4
13C label
15N label
Alanine
Pyruvate
ALT = GPT
BPTES-NP
NAD(P)+
NAD(P)H
GDH
GLS1
α-KG
Glutamate
α-KG AT
Glutamine
Glutamate
M+6
M+5
M+7
M+5
Aspartate
Oxaloacetate
M+5
M+4
α-KG AT
(such as AAT = GOT)
