# Supplementary material for: Upregulation of the Glutaminase II Pathway Contributes to Glutamate Production upon Glutaminase 1 Inhibition in Pancreatic Cancer
Source: Proteomics. 2019 Aug 1;19(21-22):1800451. doi: 10.1002/pmic.201800451 (PMC6851409; doi:10.1002/pmic.201800451)
Supplement: Supplementary file 2 — Supporting Information [file PMIC-19-na-s001.pptx]

## Slide 1
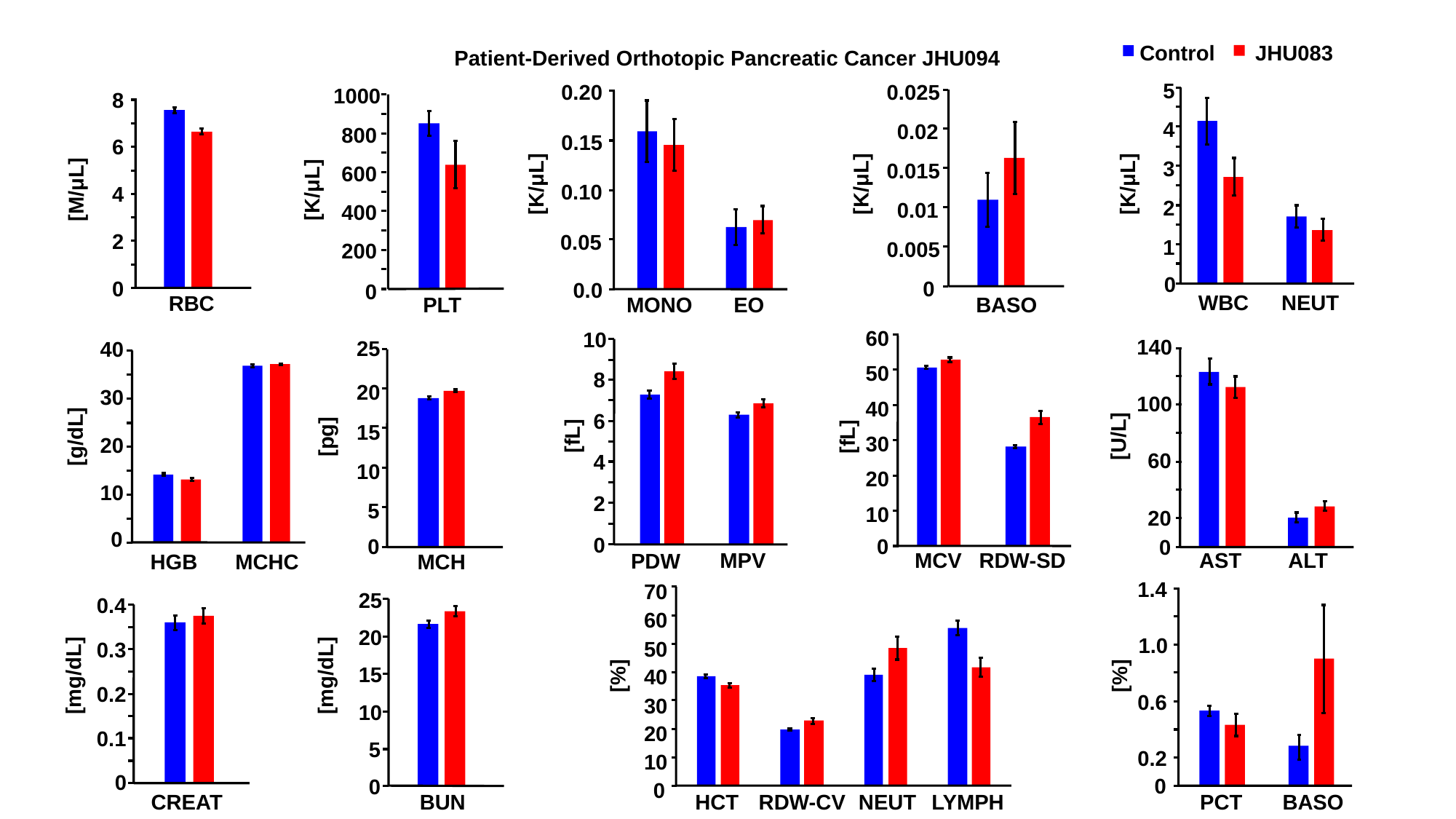

Control
JHU083
Patient-Derived Orthotopic Pancreatic Cancer JHU094
5
4
3
[K/μL]
2
1
0
WBC
NEUT
0.20
0.15
[K/μL]
0.10
0.05
0.0
MONO
EO
0.025
0.02
0.015
0.01
0.005
0
BASO
[K/μL]
1000
800
600
[K/μL]
400
200
0
PLT
8
6
[M/μL]
4
2
0
RBC
40
30
[g/dL]
20
10
0
HGB
MCHC
10
8
6
[fL]
4
2
0
MPV
PDW
60
50
40
[fL]
30
20
10
0
MCV
RDW-SD
140
25
20
[pg]
15
10
5
0
MCH
100
[U/L]
60
20
0
AST
ALT
1.4
1.0
0.6
0.2
0
PCT
BASO
70
25
20
15
10
5
0
BUN
0.4
60
50
0.3
[mg/dL]
[mg/dL]
[%]
[%]
40
0.2
30
20
0.1
10
0
0
CREAT
HCT
RDW-CV
NEUT
LYMPH
