# Supplementary material for: Upregulation of the Glutaminase II Pathway Contributes to Glutamate Production upon Glutaminase 1 Inhibition in Pancreatic Cancer
Source: Proteomics. 2019 Aug 1;19(21-22):1800451. doi: 10.1002/pmic.201800451 (PMC6851409; doi:10.1002/pmic.201800451)
Supplement: Supplementary file 3 — Supporting Information [file PMIC-19-na-s002.docx]

**Supporting Information**

**Materials and Methods**

**Animal Studies and Drug Treatment *in vivo***

Patient-derived pancreatic ductal adenocarcinoma tumors (JHU094) were used to generate the patient-derived orthotopic pancreatic cancer model. Tumors were obtained from the PancXenoBank of the Johns Hopkins School of Medicine Department of Pathology in accordance with Johns Hopkins University Institutional Review Board protocol NA_00001584. In order to retain their *in vivo* characteristics ^[^[^1^](#_ENREF_1)^]^, tumors were first subcutaneously implanted into male Foxn1^nu^ athymic nude mice (Envigo) to generate xenograft tumors. Once the xenograft tumors reached an approximate volume of 100 mm^3^, the tumors were harvested to generate orthotopic pancreatic tumors. Specifically, 100 mm^3^ tumors were cut into 2 mm^3^ pieces and were coated in a 50% (vol/vol) mixture of matrigel (Thermo-Fisher Scientific) in Dulbecco’s Modified Eagle’s Medium (DMEM, Thermo-Fisher Scientific) for orthotopic implantation. Orthotopic implantations consisted of surgical insertion of a 2 mm^3^ tumor piece into a small pocket in the pancreas of male Foxn1^nu^ athymic nude mice. The surgical incision was closed using an 8-0 nylon microfilament suture (Ethicon).

Tumor weights of Day 0 (the starting treatment day) were assessed from twelve mice who were euthanized at Day 0 and whose tumors were extracted from the pancreas and weighed. We found that that it takes 28 days (four weeks) for the tumor to reach 50-100 mg.

Four weeks after orthotopic tumor implantation into the pancreas, for targeting the glutaminase 1 (GLS1) pathway, one group of eight mice received 54 mg/kg (1.2 mg BPTES in 100 μL of nanoparticles per mouse) of BPTES-nanoparticles (BPTES-NP) by intravenous injection every three days for a total of six injections over 16 days. The other group of eight mice received 100 μL per mouse of blank-nanoparticles (Blank-NP) by intravenous injection on the same schedule as previously described ^[^[^2^](#_ENREF_2)^]^.

For targeting global glutamine metabolism using the glutamine antagonist JHU083, twenty mice bearing orthotopic pancreatic tumors were randomly divided into two groups. One group of ten mice received 1 mg/kg (0.022 mg of JHU083 in 100 μL of vehicle control per mouse) of JHU083, by intraperitoneal injection (IP) five days per week for three weeks. The other group of ten mice received 100 μL of vehicle control by IP injection on the same schedule. The vehicle control consisted of 95% (vol/vol) HEPES buffered saline (Millipore Sigma) in ethanol (Pharmco-AAPER).

Tumors were then excised for weighing and BPTES-NP and Blank-NP treated tumors were subjected to metabolomics analysis. Cardiac puncture was also performed and the blood was assessed for toxicity by the Johns Hopkins School of Medicine Department of Molecular and Comparative Pathobiology.

For xenograft models, 5 x 10^6^ P198 shGTK-KD or shControl P198 cells per tumor were suspended in a 50% (vol/vol) mixture of matrigel in DMEM. The cells were then injected subcutaneously into male Foxn1^nu^ athymic nude mice. Tumor volume was monitored using Traceable^®^ Digital Caliper (Fisher Scientific) over the course of 52 days.

**Metabolomics Analysis of Patient-Derived Orthotopic JHU094 Tumors *in vivo***

At the end of the *in vivo* treatment described above, mice were injected via intraperitoneal (IP) injection, three injections 15 minutes apart, with 100 μL of 100 mM sterile-filtered ^13^C_5_^15^N_2_-glutamine (m+7, Millipore Sigma) in phosphate buffered saline (PBS, Thermo-Fisher Scientific) in order to trace glutamine metabolism *in vivo* as previously described ^[^[^2^](#_ENREF_2)^]^. Tumors were harvested, snap frozen and homogenized in liquid nitrogen. Then the tumors were subjected to metabolic extraction using a 1:2:0.8 chloroform:methanol:LC-grade water solution as described in our previous study ^[^[^2^](#_ENREF_2)^]^. The extracted metabolites were re-suspended in 50% (vol/vol) HPLC-grade acetonitrile (Millipore Sigma) in mass-spectroscopy (MS)-grade water and metabolomics data were acquired using an Agilent 6545 Quadrupole –Time-of-Flight (Q-TOF) mass spectrometer with an Agilent 1260 High-Performance Liquid Chromatography (HPLC)-autosampler system and normalized according to protein concentration.

For data acquisition using the Q-TOF mass spectrometer, 2 μL of sample solution was drawn by an Agilent 1260 HPLC-autosampler system at 4°C. The sample solution was then subjected to reverse phase liquid chromatography for 50 minutes using a 0.1% formic acid (Thermo-Fisher Scientific) in MS-grade water mobile aqueous phase and 98% HPLC-grade acetonitrile with 0.1% formic acid mobile organic phase. The mobile phase gradient began with 100% mobile aqueous phase for first 6 minutes followed by gradual transition to a 70% mobile aqueous phase at 15 minutes and a 50% mobile aqueous phase at 19 minutes. Then, the mobile phase gradient gradually transitioned to 100% organic phase at 35 minutes and remained at 100% organic phase until the end of the runtime. The flow rate gradient began at 0.15 mL/min for the initial 27 minutes followed by gradual transition from 0.15 mL/min to 0.30 mL/min until reaching 0.30 mL/min at 47 minutes. After 47 minutes, the flow rate transitioned from 0.30 mL/min to reach 0.15 mL/min at 50 minutes. A Discovery® HS F5 HPLC Column (3μm particle size, L × I.D. 15 cm × 2.1 mm, Millipore Sigma) and a compatible guard column (Millipore Sigma) were used.

The mass spectrometer was set at the following parameters: 10 L/min gas flow, 2 μL injection volume, 45 psig nebulizer pressure, 65 V skimmer, 3500 V capillary voltage, 750 V octopole RF peak, 140 V fragmentor, and 325°C gas temperature. Positive and negative mode MS data were acquired with a mass range of 50-1700 *m/z* with a scan rate of 1.5 spectra/s. For both positive and negative modes, MS/MS data were acquired with an MS scan rate of 8 spectra/s, an MS/MS scan rate of 4 spectra/s, at collision energy of 20V, and a mass range of 40-1700 *m/z*. Reference mass calibrants were delivered using an isocratic pump for the entire duration of the acquisition.

LC-MS metabolomics data were analyzed using in-house compound standards, MS/MS fragmentation database, Agilent Mass Profiler Professional (MPP), Agilent MassHunter, and Agilent Qualitative and Quantitative Analysis Software packages. These software packages provide identification and quantification of the metabolic profile of the tumor samples. Additional metabolomics analysis was conducted using a 600 MHz NMR. We identified ^1^H NMR peaks using the Mnova software package (Mestrelab Research) and our own compound database. Peak areas were normalized using a known concentration of trimethylsilyl propionic acid standard (Millipore Sigma). Metabolic pathways were established using known biochemical relationships from the following well-cited databases: HMDB ^[^[^3^](#_ENREF_3)^]^, HumanCyc ^[^[^4^](#_ENREF_4)^]^, KEGG ^[^[^5^](#_ENREF_5)^]^, and Reactome ^[^[^6^](#_ENREF_6)^,^ [^7^](#_ENREF_7)^]^.

**Lentiviral Transduction of Pancreatic Cancer P198 Cells**

MISSION® shRNA Lentiviral Transduction Particles carrying shGTK (TRCN0000035337) or shControl (SHC001V) vector (Millipore Sigma) were obtained and used to transduce pancreatic cancer P198 cells (Johns Hopkins University PankXenoBank, IRB NA_00001584). Cells were plated at a density of 20,000 cells/mL in DMEM containing 10% (vol/vol) fetal bovine serum (FBS, Millipore Sigma) and 1% (vol/vol) penicillin-streptomycin (Pen-Strep, Millipore Sigma) and allowed to incubate overnight. The next day, 2 μg/mL polybrene (Millipore Sigma) in renewed media was added. Then, the cells were infected with lentivirus carrying the shGTK or shControl vector at a concentration of 400,000 transducing units/mL. After incubation at 37°C in 5% CO_2_ and 95% air (vol/vol) for 24 hours, the cell culture media were replaced with fresh, pre-warmed media without polybrene and transduction particles. Forty-eight hours after transduction, the media were replaced with fresh culture media containing 1μg/mL puromycin (Millipore Sigma) as predetermined for this cell line using a kill curve to select the successfully transduced cell population. Puromycin-resistant clones were then assessed for GTK expression by western blot. Cell media were replaced with puromycin-containing media every three days in order to maintain the phenotype of the successfully transduced cells. Successful clones were then used in the following experiments.

**Pancreatic Cancer P198 Drug Treatment *in vitro***

Pancreatic cancer P198 shGTK-KD and P198 shControl cells were plated in multiple 24 well plates at a density of 20,000 cells/mL in DMEM containing 10% (vol/vol) FBS, 1% (vol/vol) Pen-Strep, and 1 μg/mL puromycin and left to adhere overnight in an incubator at 37°C in 5% CO_2_ and 95% air (vol/vol). The following day, cell groups were treated with 10 μM BPTES (Millipore Sigma) in DMSO (Millipore Sigma) or DMSO vehicle control. Cell number and viability were assessed using a Vi-Cell XR™ Cell Viability Analyzer (Beckman Coulter) with the following parameters: 14.5 micron minimum diameter, 50.0 micron maximum diameter, 80% cell brightness, 100% cell sharpness, 75% viable cell spot brightness, 12% viable cell spot area, 0.000 minimum circularity, 100 images, 1 aspirate cycle, 3 trypan blue mixing cycles, and low decluster degree. Cells were detached from culture plates and placed into a Vi-Cell XR™ Cell Viability Analyzer tube prior to analysis.

**Western Blot Analysis**

PBS-washed cells were lysed in lysis buffer containing M-Per® Mammalian protein extraction reagent (Thermo-Fisher Scientific) and a protease inhibitor solution (Thermo-Fisher Scientific) and were then harvested for protein concentration. Total protein concentration for each sample was detected using a bicinchoninic acid (BCA) method (Pierce) and then quantified relative to a serially-diluted concentration of Bovine Serum Albumin (Thermo-Fisher Scientific) and measured at 562 nm using a FilterMax F5 microplate reader. 30 μg of protein were added to 25 μL Laemmli buffer (Bio-Rad) containing 2% β-mercaptoethanol (Bio-Rad) and were then heated for five minutes at 95°C. Proteins were separated on 10% Mini-PROTEAN TGX Precast Protein Gels (Bio-Rad) using gel electrophoresis at 100 V in a running buffer containing water, 10X Tris-Glycine, and 10% SDS. Proteins were then blotted onto a nitrocellulose membrane (Thermo-Fisher Scientific) using the iBlot Blotting System (Thermo-Fisher Scientific) and subsequently washed using TBST for five minutes in order to remove remaining gel. TBST was then removed and 5% filtered non-fat milk was added for blocking. After one hour, the blocking solution was replaced by rabbit anti-human GTK antibody (Thermo-Fisher Scientific) in 5% non-fat milk in a 1:1000 ratio. The membrane with primary antibody was left overnight on a cold shaker. The following day, the membrane was washed with TBST for 3 times every 5 minutes. The horseradish peroxidase goat anti-rabbit secondary antibody (Bio-Rad) in filtered 5% non-fat milk in a dilution ratio of 1:2000 was then added into the membrane. After one hour of incubation under gentle shaking the secondary antibody was removed and the membrane was washed with TBST for 3 times every 5 minutes. The membrane was then coated with ECL western blotting detection reagents (GE Healthcare) at a 1:1 ratio and subsequently developed using a ChemiDoc XRS+ Imaging System (Bio-Rad). Post-development, the membrane was stripped with stripping buffer (Thermo-Fisher Scientific) and blocked with 5% filtered non-fat milk for one hour. Horseradish peroxidase conjugated anti-α-tubulin antibody (Rockland Immunochemicals) at a 1:2000 dilution was then added to membrane. The membrane was then washed and covered with ECL western blotting detection reagents and developed using a ChemiDoc XRS+ Imaging System to assess α-tubulin levels.

**Statistical Analysis**

Values are reported as the mean ± SEM. Statistical significance was determined as NS, not significant, **p* < 0.05, **p < 0.01, ***p < 0.001 using the Student’s *t-*test. Experiment-specific statistical details, including the number of samples per experiment, can be found in the accompanying figure legends.

**Supplemental Figure Titles and Legends**

**Figure S1. Metabolomics Identification and Analysis of Glutamate Production in Patient-Derived Pancreatic Cancer (JHU094) Orthotopic Tumors (A) LC-MS/MS Identification of Glutamate Based on Fragmentation Pattern.** Fragmentation database of glutamate (top panel, courtesy of Agilent Technologies Personal Compound Database and Library) was used to match with the fragmentation spectrum of glutamate obtained from the samples at the same collision energy (bottom panel). Orange arrows indicate product ion mass-to-charge (*m/z*) matches between the sample and library fragmentation spectra. **(B) Illustration of the Transamination of Glutamate to α-Ketoglutarate Coupling with Other Amino Acids.** Illustration is presented as the production of labeled metabolites from ^13^C_5_^15^N_2_-glutamine (m+7). Red circles indicate ^13^C labeling, and green circles indicate ^15^N labeling. Metabolites are shown in black. Enzymes are shown in blue and are labeled as followed: AAT, Aspartate aminotransferase (synonym: GOT, Glutamate oxaloacetate transaminase); α-KG AT, α-Ketoglutarate-linked aminotransferase, ALT, Alanine aminotransferase (synonym: GPT, Glutamic-pyruvic transaminase); GDH: Glutamate dehydrogenase; GLS1, Glutaminase 1. BPTES-NP, shown in pink, is a GLS1 inhibitor encapsulated in nanoparticles.

**Figure S2. Blood Chemistry and Hematology Analysis of Mice bearing Patient-Derived Pancreatic Cancer (JHU094) Orthotopic Tumors after Treatment with Glutamine Antagonist, JHU083 (1 mg/kg Five Days a Week for Three Weeks**)**.** M/μL, millions per microliter; RBC, red blood cells; K/μL, thousands per microliter; BASO, basophils; EO, eosinophils; MONO, monocytes; NEUT, neutrophils; PLT, platelets; WBC, white blood cells; g/dL, gram per deciliter; HGB, hemoglobin; MCHC, mean corpuscular hemoglobin concentration; pg, picograms; MCH, mean corpuscular hemoglobin; fL, femtoliter; PDW, platelet distribution width; MPV, mean platelet volume; MCV, mean corpuscular volume; RDW-SD, red cell distribution width standard deviation; U/L, units per liter; ALT, alanine aminotransferase; AST, aspartate aminotransferase; mg/dL, milligram per deciliter; CREAT, creatinine; BUN, blood urea nitrogen; %, percentage of HCT, hematocrit; LYMPH, lymphocytes; PCT, procalcitonin; RDW-CV, red cell distribution width-coefficient of variation. Data are shown as mean ± SEM (n = 10 for vehicle control and n = 8 for JHU083 treated group).

**References**

[1] D. M. Walters, J. B. Stokes, S. J. Adair, E. B. Stelow, C. A. Borgman, B. T. Lowrey, W. Xin, E. M. Blais, J. K. Lee, J. A. Papin, J. T. Parsons, T. W. Bauer, PloS one 2013, 8, e77065.

[2] A. Elgogary, Q. Xu, B. Poore, J. Alt, S. C. Zimmermann, L. Zhao, J. Fu, B. Chen, S. Xia, Y. Liu, M. Neisser, C. Nguyen, R. Lee, J. K. Park, J. Reyes, T. Hartung, C. Rojas, R. Rais, T. Tsukamoto, G. L. Semenza, J. Hanes, B. S. Slusher, A. Le, Proceedings of the National Academy of Sciences of the United States of America 2016, 113, E5328.

[3] D. S. Wishart, D. Tzur, C. Knox, R. Eisner, A. C. Guo, N. Young, D. Cheng, K. Jewell, D. Arndt, S. Sawhney, C. Fung, L. Nikolai, M. Lewis, M. A. Coutouly, I. Forsythe, P. Tang, S. Shrivastava, K. Jeroncic, P. Stothard, G. Amegbey, D. Block, D. D. Hau, J. Wagner, J. Miniaci, M. Clements, M. Gebremedhin, N. Guo, Y. Zhang, G. E. Duggan, G. D. Macinnis, A. M. Weljie, R. Dowlatabadi, F. Bamforth, D. Clive, R. Greiner, L. Li, T. Marrie, B. D. Sykes, H. J. Vogel, L. Querengesser, Nucleic acids research 2007, 35, D521.

[4] P. Romero, J. Wagg, M. L. Green, D. Kaiser, M. Krummenacker, P. D. Karp, Genome biology 2005, 6, R2.

[5] M. Kanehisa, S. Goto, M. Hattori, K. F. Aoki-Kinoshita, M. Itoh, S. Kawashima, T. Katayama, M. Araki, M. Hirakawa, Nucleic acids research 2006, 34, D354.

[6] G. Joshi-Tope, M. Gillespie, I. Vastrik, P. D'Eustachio, E. Schmidt, B. de Bono, B. Jassal, G. R. Gopinath, G. R. Wu, L. Matthews, S. Lewis, E. Birney, L. Stein, Nucleic acids research 2005, 33, D428.

[7] L. Matthews, G. Gopinath, M. Gillespie, M. Caudy, D. Croft, B. de Bono, P. Garapati, J. Hemish, H. Hermjakob, B. Jassal, A. Kanapin, S. Lewis, S. Mahajan, B. May, E. Schmidt, I. Vastrik, G. Wu, E. Birney, L. Stein, P. D'Eustachio, Nucleic acids research 2009, 37, D619.
